# Comment on “Risk of Sarcopenia Following Long‐Term Statin Use in Community‐Dwelling Middle‐Aged and Older Adults in Japan” by Huang et al.

**DOI:** 10.1002/jcsm.13801

**Published:** 2025-04-17

**Authors:** Jian Huang

**Affiliations:** ^1^ Clinical Laboratory Center The First Affiliated Hospital of Guangxi Medical University Nanning China

Dear Editor,

I read with great interest the article ‘Risk of Sarcopenia Following Long‐Term Statin Use in Community‐Dwelling Middle‐Aged and Older Adults in Japan’ by Huang et al. [1]. Although the study addresses a topic of significant clinical relevance, I would like to express concerns regarding two critical methodological aspects that may affect the internal validity of the findings.

First, the article reported that after propensity score matching, several variables, including age, exhibit a standardized mean difference (SMD) of exactly 0.00 (see Table 1). Such a perfect balance for a continuous variable is highly unlikely, even with state‐of‐the‐art matching techniques, and strongly suggests a potential data or calculation error. Previous research has consistently demonstrated that, in practice, residual imbalances almost invariably persist despite careful matching [2]. Greater transparency in reporting balance diagnostics, for example, by providing graphical representations of propensity score distributions such as Love plots or density plots, would allow readers to thoroughly assess the adequacy of the matching process and verify that the matching has achieved an acceptable balance between groups.

Second, the authors employed risk set sampling to construct the control group; however, the article did not provide sufficient details regarding the specific algorithm used or its potential limitations. Risk set sampling is a critical method for addressing time‐dependent confounding in longitudinal studies [3], yet its implementation can vary considerably and may introduce biases if not carefully executed. The study did not specify how subjects were selected, the exact matching procedures used when sampling with replacement or any assumptions underlying the method. A detailed description of the risk set sampling algorithm, along with a discussion of its limitations and any potential impact on the study's conclusions, is essential for evaluating the robustness and reproducibility of the analysis.

These points warrant further clarification, as they have significant implications for the interpretation of the study's results.

## Conflicts of Interest

The author declares no conflicts of interest.
